# Stratus Not Altocumulus: A New View of the Yeast Protein Interaction Network

**DOI:** 10.1371/journal.pbio.0040317

**Published:** 2006-09-19

**Authors:** Nizar N Batada, Teresa Reguly, Ashton Breitkreutz, Lorrie Boucher, Bobby-Joe Breitkreutz, Laurence D Hurst, Mike Tyers

**Affiliations:** 1 Samuel Lunenfeld Research Institute, Mount Sinai Hospital, Toronto, Canada; 2 Department of Biology and Biochemistry, University of Bath, Bath, United Kingdom; 3 Department of Medical Genetics and Microbiology, University of Toronto, Toronto, Canada; Johns Hopkins University School of Medicine, United States of America

## Abstract

Systems biology approaches can reveal intermediary levels of organization between genotype and phenotype that often underlie biological phenomena such as polygenic effects and protein dispensability. An important conceptualization is the module, which is loosely defined as a cohort of proteins that perform a dedicated cellular task. Based on a computational analysis of limited interaction datasets in the budding yeast *Saccharomyces cerevisiae,* it has been suggested that the global protein interaction network is segregated such that highly connected proteins, called hubs, tend not to link to each other. Moreover, it has been suggested that hubs fall into two distinct classes: “party” hubs are co-expressed and co-localized with their partners, whereas “date” hubs interact with incoherently expressed and diversely localized partners, and thereby cohere disparate parts of the global network. This structure may be compared with altocumulus clouds, i.e., cotton ball–like structures sparsely connected by thin wisps. However, this organization might reflect a small and/or biased sample set of interactions. In a multi-validated high-confidence (HC) interaction network, assembled from all extant S. cerevisiae interaction data, including recently available proteome-wide interaction data and a large set of reliable literature-derived interactions, we find that hub–hub interactions are not suppressed. In fact, the number of interactions a hub has with other hubs is a good predictor of whether a hub protein is essential or not. We find that date hubs are neither required for network tolerance to node deletion, nor do date hubs have distinct biological attributes compared to other hubs. Date and party hubs do not, for example, evolve at different rates. Our analysis suggests that the organization of global protein interaction network is highly interconnected and hence interdependent, more like the continuous dense aggregations of stratus clouds than the segregated configuration of altocumulus clouds. If the network is configured in a stratus format, cross-talk between proteins is potentially a major source of noise. In turn, control of the activity of the most highly connected proteins may be vital. Indeed, we find that a fluctuation in steady-state levels of the most connected proteins is minimized.

## Abstract 2

Analysis of multi-validated protein interaction data reveals networks with greater interconnectivity than the more segregated structures seen in previously available data. To help visualize this, the authors draw comparisons between continuous stratus clouds and altocumulus clouds.

## Introduction

One of the advantages of a systems biological approach to understanding the relationship between genomes and phenotypes is that it permits conceptualization of intermediary levels of organization, which may not be evident from more-focused studies [1–3]. Although some of these levels can be objectively defined (e.g., cliques [4–6] and motifs [7,8]), others attempt to capture novel levels of organization in a more subjective fashion. Increasingly, biological network organization is viewed as “modular.” The underlying notion behind the idea of a module is that certain proteins function along with certain partners in a manner that renders the collections of proteins an entity in itself [9]. A simple example is the alpha and beta chains of hemoglobin that combine into a functional tetramer with cooperative oxygen-binding properties. Modules need not be stable complexes [10–13], however. One might also talk of modularity in signaling pathways, such as the bacterial chemotaxis pathway [14] or the yeast mating pathway [9]. That modules can be treated as an entity might be defended on the basis that the proteins in the same module have a similar knockout phenotype (i.e., if one is essential, all are essential; if one is nonessential, all might be nonessential), that they are phylogenetically correlated (i.e., if one is present in a given species, all are present, and vice versa) [15] or, possibly, on the basis that the constituent proteins are co-expressed.

One problem with the module concept, however, is that it is not clear where to set the boundary of the module; this nebulous aspect limits the objective definition of a module. Importantly then, Maslov and Sneppen [16] have argued that the hub proteins, i.e., those with many interactions, tend not to interact with other hub proteins, but rather prefer to interact with lowly connected proteins. This apparent tendency, termed “anticorrelation.” helps to delimit modules because the reduced interaction between hubs results in dense sub-networks that are distant from and sparsely connected to other dense sub-networks. In other words, the dense subparts are the modules. Bacterial metabolic networks often exhibit just this sort of physical modularity [14,15].

It does not follow from the above that all hub-centered parts of the network need function in the same manner. Han et al. [17], for example, contend that hubs are of two varieties, “date” and “party,” which can be distinguished on the basis of interaction partner co-expression patterns. Hubs that are co-expressed with many of their interaction partners are called “party hubs,” as all members are either present or absent together. Most protein complexes exhibit this property to some degree. In contrast, hubs that show no correlated expression with their binding partners are called “date hubs,” as in a series of two-partner dates. It has been suggested that the two categories of hubs may be intimately related to modules. Removal of date, but not party, hubs shatters the network into many smaller components, suggesting a unique role for date hubs in global network topology and, possibly, resilience to genetic disturbances [17]. Because sub-network fragments formed upon deletion of date hubs are functionally more homogeneous than fragments formed upon deletion of party hubs, it appears that party hubs reside within single modules that perform biologically discrete tasks, whereas date hubs mediate communication between different modules [17].

Two properties of hub proteins thus relate to and help define this hub-centric view of modularity in protein interaction networks: the lack of contact between hubs (i.e., anticorrelation) and the date/party hub distinction. Understanding whether these distinctions are viable is important not least because date and party hubs might be differently important in therapeutic intervention [17]. Moreover, if we are to understand the basis of dispensability of proteins, for example, then it is important that our classification of network structures reflects biological reality. Unfortunately, it is currently unclear whether these distinctions are real and helpful.

The propensity for anticorrelation is strongly influenced by the choice of dataset [16,18–20]. In the original anticorrelation analysis, Maslov and Sneppen [16] used a high-throughput (HTP) two-hybrid dataset of Saccharomyces cerevisiae protein interactions [21]. A subsequent re-analysis [18], however, suggested that exclusion of prolific baits from the dataset reduced the suppression of links between highly connected proteins, suggesting that degree anticorrelation was most likely an artifact of the particular two-hybrid dataset. In yet a third analysis, degree anticorrelation was still present when a more reliable subset of two-hybrid data (known as the Ito “core” data) in conjunction with additional two-hybrid data [22] was re-analyzed [20]. In contrast, a recent study [19] using data from the DIP (Database of Interacting Proteins) database [23] and affinity purification studies [10,21], found that the average degree of nearest neighbors is independent of node degree. Moreover, the suppression of interaction among hubs is also inconsistent with the observation that essential hubs, which tend to be highly connected, are more likely to interact with other essential hubs [24,25]. A proposed functional interpretation of anticorrelation is also tenuous. In particular, it has been argued that anticorrelation minimizes cross-talk between the highly connected proteins and thereby insulates each module from adventitious activation [16]. It is not, however, obvious that this need be so: if hub–hub suppression is a primary means to suppress “cross-talk,” then network structure alone should provide a passive mechanism to prevent unwanted communication between modules. By passive mechanism, we mean that because proteins in one module do not have binding sites for proteins in another module, no unwanted interaction can take place. Why then, we should ask, do cells use active co-localization and co-regulation of interacting partners extensively as a means to spatially regulate cellular processes [26–28]?

To circumvent the high error rate problem with HTP interaction data, all extant data have recently been distilled into a dataset of approximately 2,500 S. cerevisiae protein interactions, termed the “Filtered Yeast Interactome” (FYI) dataset [17]. This dataset was used to originally formulate the distinction between party and date hubs [17]. A strong argument that the date/party distinction is meaningful is that it holds both for partitioning based on expression correlation and, independently, for informational entropy of the hub neighborhood, which measures the localization concordance of proteins in the vicinity of hubs [17]. Thus, partners of date hubs exhibit more heterogeneous localizations than those of party hubs. However, one significant drawback with the FYI dataset is that it reflects only a very limited sub-fraction of all protein–protein interactions, probably less than 10% of the total. That the dataset may not be representative is suggested by the finding that, whereas biological networks are believed to be scale free [1], the FYI dataset used by Han et al. [17] is not scale free [29]. In addition, the evidence for co-expression of the party hubs with their binding partners was based on only a limited number of expression studies [17].

Since the publication of the above studies, three new, large-scale S. cerevisiae interaction datasets have become available: two from proteome-wide mass spectrometry–based screens [12,13] and one from comprehensive curation of the primary yeast literature [25]. The latter set of literature-curated (LC) data is of particular importance because interactions reported in the primary literature tend to be reliable because domain expertise, additional independent controls, prior contextual supporting information, and peer review reduce the likelihood of false positives. Here, using the LC data from five major interaction databases (BIND [30], BioGRID [25], DIP [23], MINT [31], and MIPS [32]) and all published HTP interaction data [10–13,21,22,33], we generated a large multi-validated dataset of 9,258 S. cerevisiae protein interactions among 2,998 proteins, which we termed the “high confidence” (HC) network. The minimum criterion for inclusion in the multi-validated dataset is that the relevant interaction was independently reported at least twice. We use the HC dataset to re-examine the issue of global organization of intermodule connectivity [16] and the generality of the date/party hub phenomenon [17] .

## Results

### Network Layout Suggests Different Global Organization

**Figure 1:**
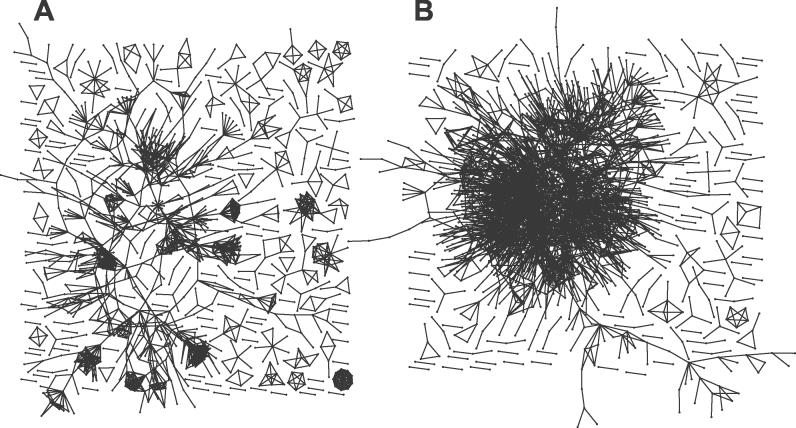
Comparison of Network Topology (A) Layout of FYI network [17]. (B) Layout of HC^fyi^ network. The small size and fragmented nature of FYI as compared to HC^fyi^ are evident: FYI contained 160 disconnected components with 57% in the main component, whereas HC^fyi^ contained 37 disconnected components with 93% in the main component. The global organization of FYI network is that of dense local regions that are sparsely interconnected (altocumulus structure), whereas that of the HC^fyi^ is densely interconnected overall, suggestive of extensive coordination and dependencies among diverse processes (stratus structure).

To allow direct comparison to the FYI network, we used a framework of all FYI proteins, to which interactions were added from the HC dataset to yield the HC^fyi^ dataset (see Methods). As the methods to create the FYI and HC datasets [17] were similar, it was not surprising that over 91% of the interactions in FYI were also present in HC^fyi^. However, as three additional large datasets were used to assemble HC [12,13,25], 51% of the HC^fyi^ interactions were not present in the FYI network. The qualitative global topologies of the FYI and HC^fyi^ networks were markedly different. The FYI network was composed of 160 unlinked sub-networks and thus highly fragmented with sparsely linked dense sub-networks in the main component, whereas the HC^fyi^ network contained one massive cohesive network bearing 93% of all proteins, accompanied by only 37 small satellite networks (Figure 1A and 1B). The most striking difference between the two networks was the suppression of connectivity among hubs in FYI; this result is encapsulated in the degree anticorrelation pattern observed previously for a HTP network [16].

### Global Degree Correlation Suggests No Bias against Hub–Hub Interactions

**Figure 2:**
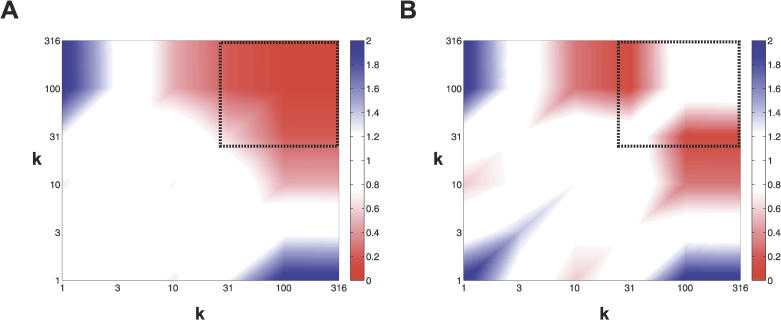
Global Degree Correlation Suggest Bias against Hub–Hub Interactions Proteins in either a combined HTP dataset (A) or the full HC dataset (B) were binned logarithmically by connectivity (with two bins per decade), and interactions among nodes in each of these bins were computed. The ratios of observed over the expected number of interactions are shown; the *x*- and *y*-axes both represent connectivity. Degree correlation profiles were computed as described in the Methods section; expected values were computed by generating 50 random networks generated via edge-swapping procedure as described [16]. All colored regions are statistically significant (standard deviation greater than or equal to ±3, representing *p* < 0.01), with enrichment highlighted in blue and depletion in red; ratios colored in white do not deviate significantly from unity. Areas bounded by rectangles represent interaction space between hubs, defined as the degree threshold exceeded by 10% of the hubs. This threshold was 18 for HTP and 21 for HC.

Degree correlations were computed as described [16] for the HC network and for a HTP network created from five large-scale datasets [10,11,21,22,33] (see Methods). Proteins were grouped into connectivity classes, and the number of interactions between each class determined. Random networks were generated using the original network as a seed by the edge-swapping method as described [16] in order to calculate the expected mean and the variance of means of interactions in each bin. On the assumption that the means were distributed normally, *z*-score normalization was performed to determine statistical significance. Connectivity of each interaction partner was plotted, and the region pertinent to modularity, i.e., connections between highly connected nodes, was bounded by a rectangle (Figure 2). The HTP network showed a clear degree anticorrelation pattern as reported previously [16] (Figure 2A). However, no such tendency was observed for the HC network: the observed fraction of interactions in the hub–hub region was no different from that expected for a scale-free network with the same degree distribution (Figure 2B).

Might this just be a peculiar property of the HC network? To investigate this possibility, we established two new datasets, called HC^m^ and HC^h^. The HC^m^ dataset consisted of only those interactions from HC that were validated by at least two different methods, and the HC^h^ dataset consisted of only those interactions from HC that were multi-validated via HTP methods only. In both of these new datasets, we again found no evidence for anticorrelation (Figure S1). As a further check, we examined the trend in hub–hub interaction suppression as we imposed ever-more stringent definitions on what constitutes a hub. For HTP, as the hub threshold was increased, the suppression became stronger, consistent with previous assertions of hub–hub suppression. However, for HC, HC^m^, and HC^h^ as the hub threshold was increased, the suppression became weaker and eventually vanished. Thus in the HC networks, there was no tendency for hubs to avoid interacting with other hubs.

This conclusion was further supported by analysis of protein essentiality. It is known that a protein is more likely to be essential if it has many protein interactions [34]. Hubs that show more interactions with fellow hubs might therefore be more likely to be essential than hubs with fewer such connections. In the HC dataset we found that this was indeed the case (mean number of hub connections for nonessential hubs = 11.15 ± 0.58, *n* = 118, for essential hubs = 14.1 ± 0.47, *n* = 189, Mann Whitney U test, *p* = 0.0002). Employing the residuals of the regression of log_10_-transformed connectivity against the number of hub–hub interactions, we additionally found that the above result held when allowing for differences in absolute number of connections (Mann Whitney *U* test, *p* = 0.01). We surmise both that hubs with more hub interactions are more likely to be essential and that hub–hub interaction must in turn be a real phenomenon.

### Fraction of Hub–Hub Interactions Reduce with Experimental Scale

**Figure 3:**
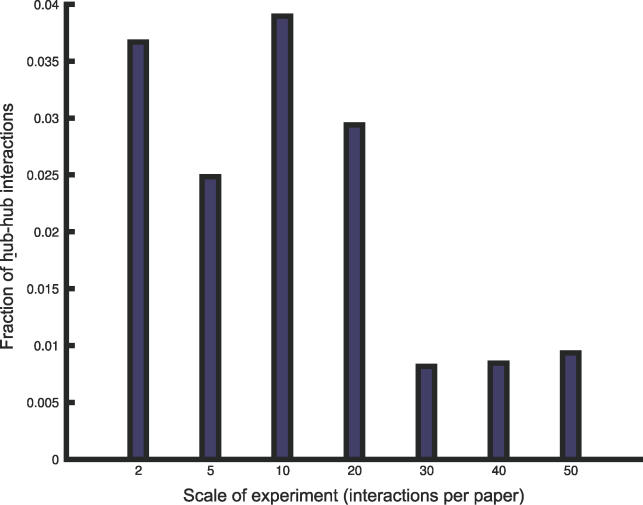
Fraction of Hub–Hub Interactions Reduce with Scale of Experiments Protein interactions from the LC dataset were separated into subgroups based on the number of interactions reported in the same publication, which was taken as a proxy for experimental scale. For each sub-network, hubs were defined as protein with connectivity exceeding the 90th percentile. The strong negative correlation (*r* = −0.85, *p* < 0.01, Spearman) indicates that as the size of the screen increases, bias against hub–hub interactions begins to appear.

To examine the assertion that proteome-wide interaction screens may have limited capacity to discern hub–hub interactions, we partitioned the LC dataset into sub-networks according to scale. That is, curated publications were partitioned by the number of interactions reported in each (i.e., the scale of the experiment) such that the total number of interactions in each bin were approximately similar in number. For each sub-network, we defined hubs as proteins whose connectivity exceeded the connectivity of 95% of the other proteins in that sub-network. By this measure, the fraction of hub–hub interactions decreased with the scale of experiment (*r* = −0.85, *p* < 0.01, Spearman rank correlation). The sharp drop beyond a scale of 20 interactions per paper indicated an onset of bias against hub–hub interactions after this point (Figure 3). This result suggests that large-scale screens have difficulty in assessing interactions among hubs (see Discussion).

### No Evidence for Date and Party Hub Distinction

The above results indicate that the apparent avoidance of hub–hub interactions is an artifact of prior data, most parsimoniously explained by potential experimental biases rather than natural selection [16]. What then of the distinction between date and party hubs? Evidence for these came originally from four sources: (1) effect of hub deletion; (2) correlated neighbor expression profiles; (3) shared localization annotation of neighbors; and (4) centrality in the genetic interaction network. Subsequently, further support came from the finding of different rates of evolution of date and party hub protein [35]. Given the different topologies of the FYI and HC^fyi^ networks, we re-examined each property in turn.

#### The HC^fyi^ network is tolerant to hub deletion.

**Figure 4:**
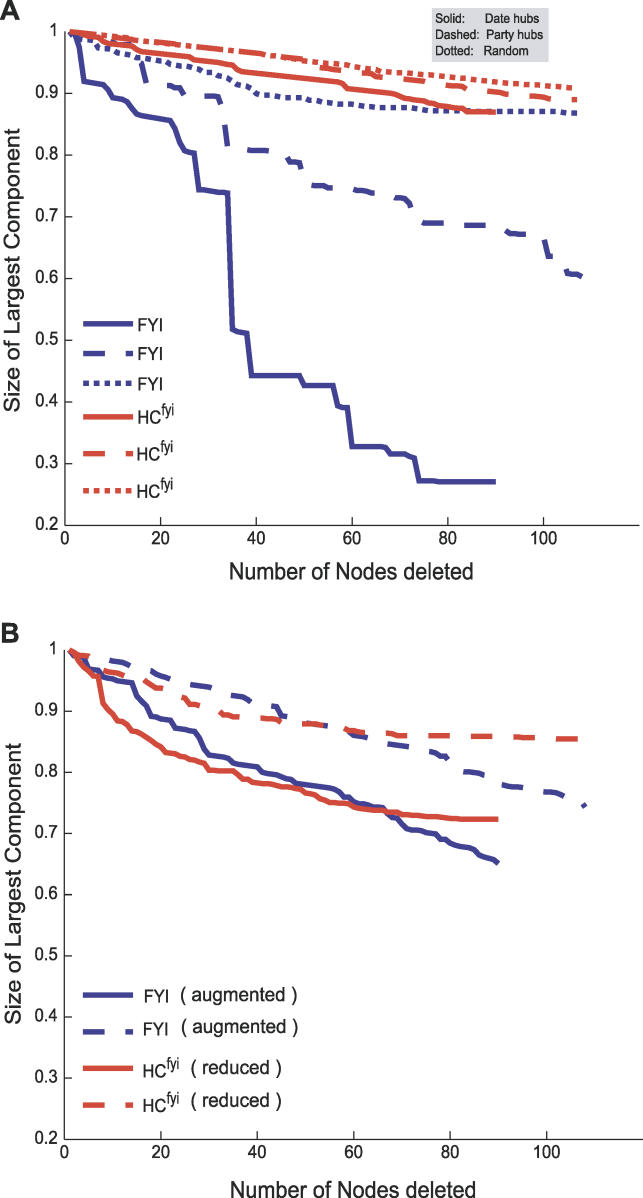
Network Tolerance to Hub Deletion The size of the largest component after deletion of hubs in the indicated networks was normalized by the initial size of the largest component. In all cases, hubs were deleted in descending order of connectivity. (A) The FYI network is sensitive to hub deletions, whereas the HC^fyi^ network is tolerant to hub deletions. (B) Topological sensitivity to node deletion can be modulated by varying the fraction of hub–hub interactions. Addition of 10% of random hub–hub interactions is sufficient to increase the tolerance of the FYI network to hub deletion (“augmented”). Deletion of 40% of interactions among hubs is necessary to increase the sensitivity of the HC^fyi^ network to hub deletion (“reduced”).

To probe the assertion that date hubs serve as intermodule linkers, we determined the effect of date and party hub deletion on the HC^fyi^ network. Note that provided the date/party distinction is a biologically meaningful property, as new interactions are integrated into an existing network, the identity and attributes of date and party hubs should not change. To measure susceptibility of HC^fyi^ network integrity to disruption, we serially deleted either the entire set of putative date hubs or the entire set of putative party hubs in descending order of connectivity, and computed the size of the largest remaining component at each step (Figure 4A). The drastic collapse of the FYI network originally observed upon deletion of date, but not party, hubs was recapitulated [17]. In contrast, in the HC^fyi^ network, neither date nor party hub deletion had any effect beyond that of random node deletion. A smaller FYI-derived network constructed only from interactions present in the LC dataset, called LC^fyi^, was similarly resistant to date and party hub deletion (Figure S2). The complete HC, HC^m^, HC^h^, and LC networks were also highly resistant to date hub deletion (Figure S2 and unpublished data).

To test whether the differences in hub–hub interactions could explain the differential sensitivity of the FYI and HC^fyi^ networks, we both added random interactions among hubs to the smaller FYI network, or deleted random hub–hub interactions from the larger HC^fyi^ network, and then repeated the deletion analysis. An increase of just 10% in random hub–hub connections to FYI dramatically increased its tolerance to hub deletion, whereas removal of 40% hub–hub connections from HC^fyi^ was required to partially increase its sensitivity to hub deletion (Figure 4B). The 40% decrease in hub–hub connections reduced the size of HC^fyi^ to the size of FYI; we note that in order to have sufficient hub–hub interactions to enable this massive level of deletion, the connectivity threshold used to define a hub in the reduced HC network was set to 50%. Network sensitivity to hub deletion is thus explained in part by small size of the FYI network and in part by bias against hub–hub interactions in HTP-derived datasets.

The marginal topological effect of date/party hub deletion on the HC^fyi^ network at first seemed to conflict with the established property that scale-free networks are sensitive to deletion of hubs [36]. However, despite the fact that FYI date and party hubs were also hubs in the HC^fyi^ or full HC networks, these were not necessarily the most connected nodes in these networks. Indeed, when we deleted the top 20% of the most-connected nodes and compared the size of the residual largest component, HC^fyi^ network topology was severely compromised (Figure S3), as observed previously [36].

#### No evidence for bimodality in expression correlation of hub partners.

A primary criterion for the distinction between date and party hubs in the FYI network is the bimodality in expression correlation of hubs [17]. This bimodality is important as it would indicate that, pooling all hubs together, the party hubs (with high co-expression) are a qualitatively distinct subclass, rather than part of a continuum. However, the statistical significance of this apparent bimodality was not determined in the original analysis [17]. We therefore used Hartigan's DIP test [37,38] to test whether the probability density of neighbor expression of date/party hubs matched a null unimodal distribution. The DIP test fits the best unimodal distribution of any shape and then scores the deviation of the observed data from this distribution. Higher DIP scores imply greater deviation from unimodality. For empirical distributions of the mean expression correlation of hubs with their neighbors computed using 25 different large expression datasets, unimodality could not be rejected for all networks tested (Table 1).

**Table 1:**
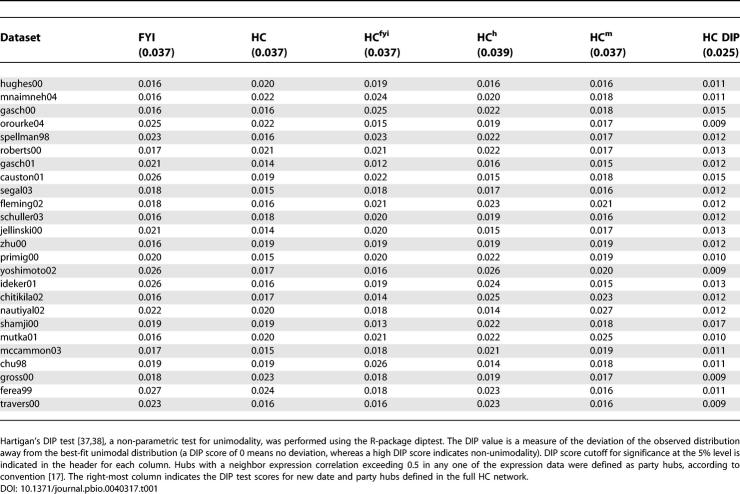
Statistical Test for Multi-Modality in the Distribution of Neighbor Expression Correlation

To determine whether distinct classes of date and party hubs exist in the full HC network, we defined the top 15% of highly connected proteins in HC as hubs, and tested whether the density of neighbor expression correlation showed bimodality across the same 25 expression datasets. The full HC network invariably exhibited a single mode (Table 1). We concluded that the putative bimodality in the small FYI network represents random fluctuations in the empirical distribution due to small sample size, rather than a meaningful partition of hubs into two distinct spatio-temporal classes. This small sample-size effect may also be exacerbated by the enrichment for interaction amongst strongly co-expressed proteins as judged by higher fractions of interactions in FYI but not in HC with shared GO annotations (Table S1).

### 

#### Localization entropy of date and party hubs.

The bimodality of neighbor co-expression is apparently also buttressed by an independent attribute, namely the differential enrichment of shared localization annotation [27] among neighbors of date and party hubs [17]. That is, neighbors of date hubs have more diverse localizations than neighbors of party hubs, as measured by information entropy [17]. The more evenly distributed the set composition, i.e. no enrichment of any character, the higher the entropy. However, this conclusion is subject to several caveats. First, spatial diversity was not normalized to account for the increased connectivity of date hubs [17]. Since the entropy measure used to assess diversity is not independent of size, neighbors of date hubs, which are more connected than party hubs, might be artificially enriched for more diverse locations by virtue of more connections. Second, cytoplasmic and nuclear localization were excluded from consideration in the FYI analysis without adequate justification [17]. When these compartments were included and/or data were appropriately normalized, date hubs in fact showed lower entropy (i.e., enrichment) for common localization in their neighbors, than party hubs in five networks (Table 2). This lack of enrichment was consistent with the absence of bimodality in the gene expression in rejecting the date/party distinction (Table 1).

**Table 2:**
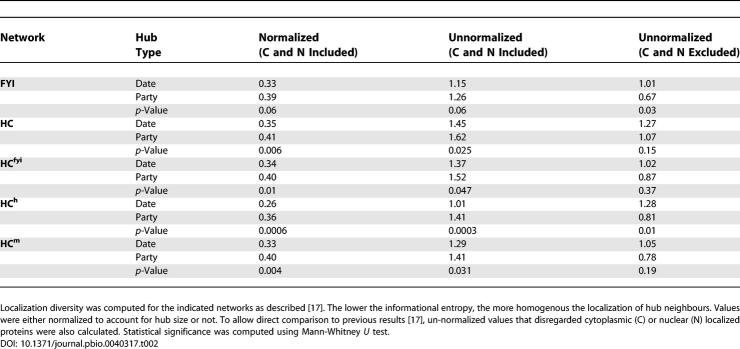
Localization Entropy Difference of Hubs

The above tests, while rejecting the hypothesis that date hubs have higher entropy, nonetheless reveal an unexpected finding, namely that the inverse correlation appeared to be true, i.e., party hubs actually had higher entropy than date hubs. This property appeared to derive from the fact that party hubs tend to be more abundant proteins. In both rich and poor media, entropy was positively correlated with abundance: in both media conditions, Pearson product moment correlation, Log(Abundance) versus Ln(Entropy) *r* ~ 0.25, *p* < 0.005, N_rich_ = 117, N_poor_ = 110. Importantly, when controlled for abundance, date and party hubs did not differ in their entropy (for HC: analysis of covariance [ANCOVA] with Ln(Entropy) predicted by date/party with Log(Abundance) of a covariable: rich media: *p* for date/party effect, 0.72, *p* for effect of abundance, 0.01; poor media: *p* for date/party effect, 0.68, *p* for effect of abundance, 0.01). Why abundance might affect entropy is unclear; however, highly abundant proteins may have more diffuse locations in the cell [26]. Indeed, the more highly abundant a protein, the more different cellular compartments it is seen in, although the effect is very weak (Spearman rank correlation, rho = 0.04, *p* < 0.02, *n* = 2,508). This effect may be real or may indicate a higher rate of misclassification for the more abundant proteins.

##### Genetic centrality of date versus party hubs.

A further distinguishing feature of FYI date hubs is their apparent propensity to exhibit more synthetic lethal interactions than either party hubs or non-hubs, suggesting a central genetic role for date hubs [17]. However, this analysis was based on a small set of approximately 1,000 synthetic lethal interactions [32]. For a more exhaustive comparison of genetic centrality of date, party, and non-hubs, we used two different sets of genetic data recently compiled from the primary literature [25], grouped into systematic methods (called HTP-GI, consisting of SGA [39,40] and dSLAM [41] genome-wide screen data) and conventional focused tests of genetic interactions (called LC-GI). On average, date hubs had more genetic interactions than party hubs or non-hubs in the LC-GI network, whereas in the HTP-GI network, date hubs actually had fewer synthetic lethal partners (Figure S4). Due to this contradictory result, we conclude that at this point, it is not possible to make an unequivocal statement that date hubs are genetically more central than party hubs. However, because HTP genetic screens are inherently non-biased, at least as regards to cellular process, whereas focused genetic interrogations are often biased towards interaction partners [25], we suspect that as the genetic network grows, the residual genetic centrality of date hubs will disappear.

##### Date and party hub proteins evolve at the same rate.

It has been claimed that data and party hub proteins evolve at different rates [35]. The logic of why this might be can be simply explained by comparison with electrical circuits. In such circuits there are sets of switches that control electrical units (e.g., light bulbs). Although the electrical switch can be rewired to control a different set of lights, the structure of the light bulb cannot be changed. Party hubs, it is then argued, form coherent modular elements (c.f., light bulbs) whose structure is fixed. Date hubs, by contrast, function as switches that can evolve to control different sets of lights. For this sort of reason, it is suggested that date hubs evolve faster than party hubs. Were this difference in evolutionary rate real (and the interpretation correct), it would suggest that, contrary to what we have identified above, there is some genuine and verifiable difference between date and party hubs.

To re-examine the relationship between rates of evolution for party and date hubs, we established a new, much larger dataset and used the new HC network to define date and party hubs, using the same criteria employed previously [17]. Naturally, the two hub classes have drastically different co-expression values as this parameter is used to define the two sets (median co-expression for date hubs = 0.05, for party hubs = 0.28). Using rates of protein evolution from the *S. cerevisiae–S. bayanus* alignments (see Methods), we found no significant difference in evolutionary rates between the two classes (median *dN* for party = 0.055, *n* = 110, for date = 0.074, *n* = 200, Mann-Whitney *U* test: *p* = 0.08). Transforming the data by natural log transformation, such that the distributions were approximately normally distributed, reinforced this lack of difference between the two classes (mean Ln(*dN*) for party = −3.2 ± 0.086, for date = −3.0668 ± 0.083, *p* = 0.27, *t*-test).

**Figure 5:**
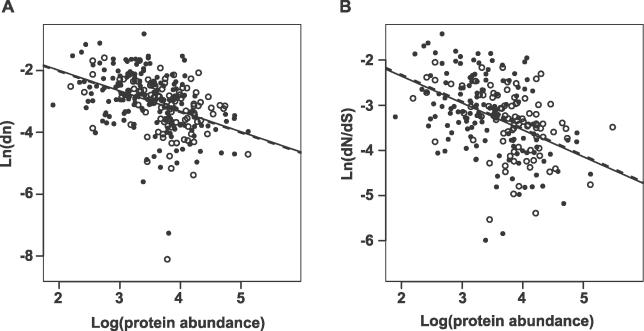
Date and Party Hubs Evolve at the Same Rate When Controlled for Protein Abundance The relationship between *dN* (A) and *dN/dS* (B) as a function of protein abundance was determined for party hubs (open circles) and dates hubs (filled circles). Best-fit lines assuming equal slopes for the two hub types are shown (party hub, dashed line; date hub, solid line).

The small residual difference between the two hub classes in the HC network was readily explained by differences in protein abundance, which is a major predictor of both rates of evolution [42] and a strong correlate to the level of co-expression (Spearman rank correlation between protein abundance [43] and co-expression: rho = 0.40, *p* < 0.0001). The difference in protein abundance between the date and party hubs was striking: the median abundance for party hubs was 6,560 molecules per cell and for date hubs was 2,740 molecules per cell (*p* < 0.0001, Mann Whitney *U* test). If we allowed for this difference, there was not even a weak tendency toward different evolutionary rates between date and party hubs (ANCOVA, Ln(*dN*), predicted by date versus party with log_10_(Abundance) as a covariate: *p* for effect of date versus party = 0.73, *p* for effect of log_10_(Abundance) < 0.001; Figure 5A). Note that in a fuller model permitting an interaction term between hub type and protein abundance, the interaction term was not significant (*p* = 0.545); hence we could not reject the null of equal slopes, and so the ANCOVA assumption of equal slopes was valid.

We further verified this result by employing *dN*/*dS* (using corrected *dS*) derived from a different species comparisons across the sensu strictu yeasts [44]. As *dN/dS* correlated very highly with the *dN* measure (Spearman rho = 0.97), it was not surprising that employing this measure did not alter the above conclusions (ANCOVA, Ln(*dN*/*dS*), predicted by date versus party with log_10_(Abundance) as a covariate: *p* for effect of date versus party = 0.72, *p* for effect of log_10_(Abundance) < 0.001; Figure 5B). Controlling again for protein abundance, we also found no evidence that essential hubs evolve slower than nonessential hubs (ANCOVA, Ln(*dN*), predicted by essential/nonessential with log_10_(Abundance) as a covariate: *p* for effect of essential/nonessential = 0.53, *p* for effect of log_10_(Abundance) < 0.001; for ANCOVA of Ln(*dN*/*dS*), *p* = 0.97 and *p* < 0.001 respectively). Dispensability was, hence, not an important variable for this analysis.

Finally, using a general linear model, we found that, controlling for abundance, in both rich and minimal media [43], co-expression rate was not a predictor of *dN,* nor *dN*/*dS:* Ln(*dN*/*dS*) predicted by date/party (*p* = 0.59), co-expression (*p* = 0.68), and log_10_(Abundance) (*p* < 0.001); Ln(*dN*) predicted by date/party (*p* = 0.81), co-expression (*p* = 0.50), and log_10_(Abundance) *p* < 0.001). From this exhaustive analysis of the larger HC dataset, we conclude that the prior claim of an evolutionary difference between date and party hubs [35] appears not to be robust.

## Discussion

In the large, multi-validated HC network generated from all extant S. cerevisiae protein interaction data, we find that the global interaction network does not show degree anticorrelation nor do hubs fall into clear date and party sub-populations based on neighbor expression correlation. More generally, we find no coherent evidence that the date/party distinction evident in the FYI network is helpful in understanding the behavior of the global protein interaction network. By contrast, we find that the number of interactions with fellow hubs is helpful in understanding the dispensability of hub proteins. Finally, we demonstrate that HTP methods appear to have difficulty in assessing interactions among hubs, as compared to more focused interaction studies.

Two possibilities might explain the observed discrepancy in degree correlation patterns in HC versus HTP networks: either the degree anticorrelation observed in HTP networks is due to a bias of HTP approaches against hub–hub interactions, or the lack of degree anticorrelation in HC is due to enrichment of essential nodes. As essentials are more connected than nonessentials [34] and essentials are more likely to be connected to other essential proteins [25], enrichment for essentials necessarily increases hub–hub interactions. To test this latter explanation, we computed degree correlation for a reduced HC network that had interactions among nonessential proteins only; however, there was no degree anticorrelation in this reduced network either (Figure S5). Our analysis suggests that degree anticorrelation derives from ascertainment bias of large-scale screens against hub–hub interactions, rather than by natural selection for physical isolation of modules previously surmised [16]. Suppression of hub–hub interactions in HTP data may arise from non-saturating coverage of interactions, perhaps as a consequence of signal suppression by partner–partner competition. It is also possible that surface masking of binding sites on highly connected proteins suppresses detection of hub–hub interactions and/or that nonspecific interactions of abundant proteins creates false hubs in HTP data.

A combination of small network size, high false-negative rate, and depletion of hub–hub interactions explains why FYI date and party hubs no longer have their defining characteristics in the HC^fyi^ or the full HC network, which are two and four times larger than the FYI network, respectively. Indeed, we demonstrate that addition of a small fraction of random hub–hub interactions to the FYI network is sufficient to increase tolerance to hub deletion, whereas removal of 40% of hub–hub interactions from the HC^fyi^ network is needed to increase susceptibility to network collapse. We also find that the distinction between party and date hubs on the basis of bimodality, localization entropy, genetic centrality, and rates of evolution does not bear scrutiny.

These results not only reject the notion of hub-centric modularity, they also suggest a new view of the yeast protein interaction network. To illustrate the differences between the old and the new views of the network, we make a simple analogy with cloud formation. In the now-standard view, in which functional modularity is thought to derive from physical modularity, the protein network is rather like altocumulus clouds, i.e., with wisps of thin cloud connecting and blue sky visible between the high-density clouds. In the new view espoused here, the network is more akin to stratus clouds, i.e., a much more dense and lumpy distribution of cloud that forms a thick cover through which blue sky cannot be seen, just varying levels of grey and white.

**Figure 6:**
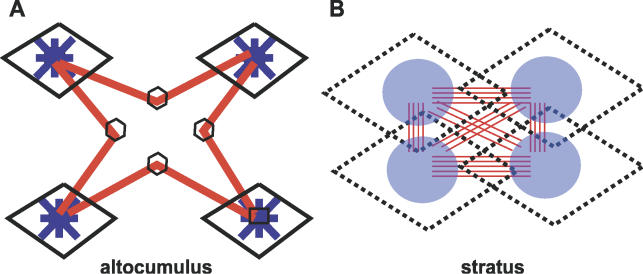
Modularity Paradigms Two representations of protein interaction network topology are shown. (A) Conventional view of functional modularity in which hub-centric modules are sparsely interconnected, i.e., a modular physical topology underlies functional modules [16,17]. (B) An integrated view in which functional modules are heavily interconnected, as supported by the large-scale organization of dense HC networks.

What do these results mean for our conception of modularity in the protein interaction network? The new stratus conceptualization has the advantage that it can accommodate two well-established facts of signal transduction networks (Figure 6). First, many proteins are multi-functional and/or take part in multiple complexes [12,45]; second, signalling pathways or modules often overlap or share sub-circuits (Figure 6B). By contrast, the hub-centric view of modularity [16] does not support participation of proteins in multiple complexes (Figure 6A). Consequently, if the global network is indeed stratus-like in nature, then the search for modules will likely not be as straightforward as finding densely connected regions or cliques.

We stress that the idea of modularity in the S. cerevisiae proteome is an eminently useful concept. Discrete functions are often carried out by protein sub-networks or something that one might like to call modules. The interactions between these modules, however, appear to meld the global network into the stratus-like whole, thereby rendering meaningful topological definition of modules harder to establish. This view is further buttressed by the stratus structure of the global genetic interaction network [39,40]. We also point out that our observations in budding yeast may or may not hold in other species. For example, prokaryotic networks appear to exhibit modularity [46] in part owing to co-transfer of genes in the same metabolic pathways via horizontal gene transfer [47], a process, for the most part, not seen in yeast.

One might reasonably suggest that the new stratus view of the cellular network could be positioned somewhere on a continuum between a homogenous network and the previously accepted discretized altocumulus model. What is the significance of repositioning of the network on this continuum away from the altocumulus model, and what, if any, is the use of the stratus metaphor? First, as we have shown, the realization that hub–hub interactions are not suppressed leads to a better understanding of knockout phenotypes, i.e., the more hub interactions a hub protein has, the more likely it is to be essential. Moreover, we should like to suggest that this new stratus view of the network is important as, like all good metaphors, it should act to redefine the issues of interest. In this instance, we suggest that we should now focus on the problem of control of protein interaction noise and cross-talk. In the altocumulus view of the network, the relatively discrete nature of the modules was thought to reflect an adaptation to minimize cross-talk that might arise from promiscuous protein–protein interactions. That the network is more stratus-like suggests, by contrast, that inappropriate cross-talk could be a major problem for the cell, while at the same time, appropriate cross-connections could be a primary basis for coordinating different aspects of cellular responses.

**Figure 7:**
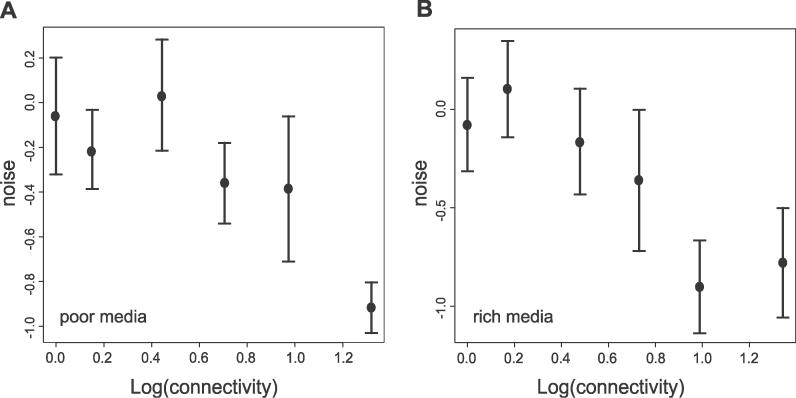
More Highly Connected Proteins Exhibit Less Noise Under both nutrient-poor (A) and nutrient-rich (B) conditions, more-connected proteins show less noise in their prevalence at the single cell level [43] when controlled for absolute protein abundance levels. For these plots, the data were split in to equal-sized bins of approximately equal connectivity. The values on the *x*-axis indicate the mean log connectivity of each bin.

Given the possibility for egregious cross-talk, we would expect to see that highly connected proteins are more tightly regulated. In this vein, we recently showed that hubs (defined in the LC set) do indeed have more phosphorylation sites, and their mRNAs have shorter half-lives [48]. Similarly, we expect that more-highly connected proteins should have less noise in their expression than less-connected ones. To address this, we considered the relationship between the variations in protein abundance in single cells on the same growth media, this being taken as a measure of gene expression noise [43]. This variation was then normalized to allow for covariance between the level of noise (i.e., the coefficient of variation) and the absolute level of protein abundance (see Methods). We then find for all datasets that the most-highly connected proteins do indeed tend to have lower levels of noise, when controlled for abundance (Table 3; Figure 7). To allow for the fact that essential genes tend more often to be highly connected and have lower noise, we repeated this analysis for nonessential genes alone, and obtained the same result (Table 3). We also controlled for growth rate of the nonessential genes and compared the *r* ^2^ values from the analysis of noise predicted by growth rate controlled for connectivity with those from noise predicted by connectivity controlled for growth rate, for the same comparison data set. We find that in six of eight cases (four networks, two growth conditions), abundance-corrected noise [43] predicted by connectivity controlled for growth rate and mRNA half-life had the higher *r* ^2^ suggesting that connectivity per se is an important variable predicting noise (unpublished data). The mean *r* for these partial correlations is −0.095, suggesting that any effect is probably relatively weak when covariance between connectivity and dispensability is allowed for.

**Table 3:**
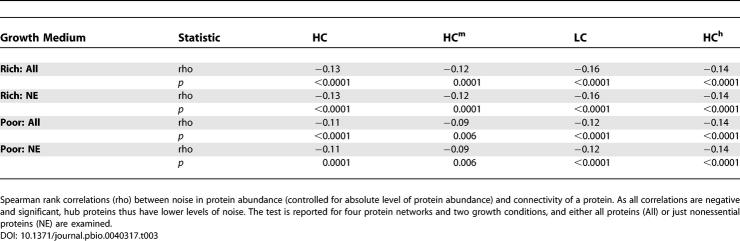
Relationship between Noise in Protein Abundance and Connectivity

The relationship between network structure and cross-talk may well have implications for understanding human disease. For instance, disease states often arise from over-expression of disease genes; salient examples include oncogenes that are over-expressed in cancer cells and the chromosomal aneuploidies that underlie many inherited syndromes. It is possible that over-expression might be especially deleterious if the protein involved is a hub with many hub interactions. Analysis of the means by which, in a stratus-like network, the control of promiscuous protein–protein interactions will, we suggest, be an important avenue for future work.

## Materials and Methods

### Protein interaction data.

A HTP dataset of 11,571 interactions among 4,474 proteins in S. cerevisiae was created from the union of five large-scale datasets [10,11,21,22,33]. An LC dataset of 11,334 interactions among 3,289 proteins was obtained from the BioGRID database (http://www.thebiogrid.org) [25,49]. The FYI dataset of 2,491 interactions among 1,375 proteins was obtained from Han et al. [17]. FYI data were made from intersecting multiple curation, HTP, and in silico–predicted interactions. The HC dataset of 9,258 interactions among 2,998 proteins and the HC^fyi^ dataset of 3,976 interactions among 1,291 proteins were derived from the overlap of all extant protein interaction datasets (i.e., all LC interaction data and all HTP interaction data, including two recent proteome-wide surveys [12,13]), as described below. For consistency checks, we generated two subsets of the HC networks: the HC^m^ dataset consisted of only those interactions from HC that were validated by at least two different methods, and the HC^h^ dataset consisted of only those interactions from HC that were multi-validated via HTP methods only.

### Generation of HC network.

The FYI network was created with an intersection method [17] in which only the interactions that were observed at least twice in various datasets were retained. As three large protein interaction datasets have become available since the construction of FYI, we therefore generated a large HC network using the same intersection approach. HC was built from LC interaction datasets housed in five interaction databases (BIND [30], MIPS [32], MINT [31], DIP [23], and BioGRID [25]), six large-scale interaction datasets [10–13,21,22,33], and an in silico–predicted dataset [50]. For all interactions detected by mass spectrometric analysis of protein complexes [10–13,21,22,33], we represented interactions in a minimal “spoke” form, i.e., as single direct interactions between bait and prey proteins [4]. After removing interactions derived from standard large-scale experiments [10,11,21,22,33] from each of the interaction databases, the remaining curated interactions from focused studies were partitioned into two sets: those that came from papers that were in at least one other curated database, and those that were from papers unique to each curated database. Interactions in the same direction, but from independent papers or different methods (if from the same paper), and interactions in the reciprocal direction were considered to be multi-validated. All singly validated interactions from the reduced curation datasets were merged and multi-validated interactions were added to the final HC network. In order to construct the HC dataset in the most conservative manner possible, we removed all interactions that occurred in 100 or more different co-purifications from the raw Gavin et al. dataset [12]. In addition, we did not include the MIPS [32] complexes dataset (as was done by Han et al. [17]) because there was no bait defined for these complexes and an exhaustive matrix model representation of all pairwise interactions over-represents these complexes (i.e., adds false positives). All network elements were thus minimal spoke model representations [4]. See Figure S6 for a schematic of how datasets were combined; a list of all interactions in the HC dataset is provided in Table S2. We caution that the intersection method of selecting HC interactions may add unexpected biases and increase the false-negative rate of the filtered dataset. For example, established interactions are often not published more than once and hence would be excluded; moreover, interactions between low-abundance proteins, while real, may not be readily repeated in different experiments and hence be eliminated from the final dataset.

### Genetic interaction data.

For comparison of genetic centrality of date, party, and non-hubs, we used two different sets of genetic interactions, grouped into HTP (genome-wide SGA and dSLAM methods, called HTP-GI) and small-scale studies curated from the literature (called LC-GI). The HTP-GI network was curated from 39 different papers [25], including two large genome-wide screens [39,40], and contained 6,103 interactions among 1,454 genes; this network consisted of only synthetic lethal/synthetic sick interactions and was depleted for essential genes because query genes are screened against the approximately 5,000 viable gene deletion strains. The LC-GI network contains 8,165 interactions among 2,689 mutants, and was curated from 3,798 publications [25]; this network contained the following classes of genetic interactions: synthetic rescue, synthetic lethality, dosage lethality, phenotypic suppression, synthetic growth defect, dosage growth defect, dosage rescue, and phenotypic enhancement. Details of these classes of genetic interactions are described elsewhere [25].

### Degree correlation profiles.

Correlations in connectivity of interacting proteins for each of the two networks were computed as described [16]. Briefly, we calculated the likelihood *P(k_0_ ,k_1_)* that two proteins with connectivity *k_0_* and *k_1_* are connected to each other, and compared it to the same quantity *P_r_(k_0_ , k_1_)* measured in the randomized version of the same network using an edge-rewiring procedure that preserves the degree distribution. Correlations in connectivity are readily apparent as systematic deviations of the ratio *P(k_0_ , k_1_)/P_r_(k_0_ , k_1_)* from unity. To reduce visual clutter and simplify interpretation, only statistically significant regions (using a threshold of plus or minus three standard deviations, representing *p* < 0.01) were reported.

### Defining hubs in networks of different sizes.

As connectivity scales with network size, it is not possible to fix a static degree cutoff to define a hub. We circumvent this problem by defining a different threshold for each network as follows: in each dataset, hubs are defined to be proteins whose connectivity exceeds the connectivity of a certain percentile of the nodes in the dataset (thresholds set at either 90% or 95% unless mentioned otherwise).

### Neighbor expression correlation.

All 25 expression data were normalized to have a mean of 0 and a standard deviation of 1, and missing data were replaced by the column mean. In all cases, Pearson correlation coefficient was used. Because of their possible unusual evolutionary selection for very high expression level and tight co-regulation under stress, genes encoding ribosomal proteins were excluded for the neighbour expression correlation analysis. Sources of expression data are given in the supporting information.

### Effect of hub deletion on network topology.

Largest network components were determined using Breadth First Search [51] and normalized to the size of the largest component prior to node deletion so that resilience of networks of different sizes can be compared. In all cases, nodes were deleted in descending order of connectivity.

### Localization diversity.

Using a large-scale localization dataset [27], neighbor localization diversity or entropy was computed as −sum(*L_ij_* * log_2_(*L_ij_*) )/ *M_j_*, where *L_ij_* is the frequency of localization *i* in set *j*. Multiple localizations were treated as additional entries (i.e., if a given protein has localization of cytoplasm and nucleus then we counted each one of these localizations) and use *K_j_* instead of *k_j_*, to denote the adjusted set size. As entropy depends on the set size, we normalized by the uniform distribution, *M_j_,* which is the maximum entropy distribution [52]. As the set size may be smaller than the total number of localizations (and consequently not all localizations could have appeared), normalization not only depends on the actual size of the set, but also on whether the set size is larger or smaller than the total number of unique localizations: if the adjusted set size, *K_j_,* is smaller than the number of possible localizations, *n,* then the maximum possible entropy is −log(*K_j_*) otherwise it is −log(*n*).

### Statistical test for bimodality.

The DIP test [37,38], a non-parametric test for testing for multimodality against the null of a unimodal distribution, was computed using the “diptest” package for R (http://www.r-project.org). The DIP statistic is the distance between the “tightest fitting” unimodal distribution function and the empirical kernel distribution, i.e., the maximal difference between the empirical kernel distribution function and the unimodal distribution function that minimizes this maximum difference. The null DIP distribution is the uniform distribution. To calculate significance, we obtained the 95% upper limits for dip from 10^6^ simulations (variable “qDiptab” in the diptest package). We then determined the best-fit curve relating the 95% critical DIP score and N the sample size, this being:

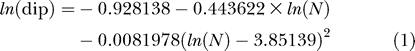
The fit between this line and simulation data has *R^2^* > 0.999. From this line we determined the critical DIP score for a given sample size.

### Relating noise to connectivity.

Recent data [43] have been presented relating the variation in protein abundance between cells grown under the same growth conditions, with two different conditions being employed. These data are reported as the coefficient of variation in abundance for many yeast proteins (the higher the coefficient of variance, the more noisy the expression of the protein). This measure is by necessity related negatively to protein abundance. To control for this, we compared the variation to the inverse of the square root of abundance (which, as expected, provides a strong linear fit). From the residuals of the regression of this inverse against the coefficient of variation, we derive a measure of noise that is independent of abundance. Positive values imply more noisy expression. We then ask whether these residuals are predicted by the level of protein connectivity. Results are given in Table 3.

### Calculating rates of evolution.

Protein evolutionary rate data were based on the alignments of S. cerevisiae and S. bayanus orthologs. Nonsynonymous divergence, *dN,* was then estimated using PAML [53] with nine free parameters used to account for codon frequencies (F3x4). Corrected measures of the number of synonymous changes per synonymous site were taken from prior analysis [44].

## Supporting Information

Figure S1 Degree Correlation for HC^h^ and HC^m^ Networks (120 KB PDF)

Figure S2 The Topology of the LC and LC^fyi^ Networks Is Not Sensitive to Deletion of Date or Party Hubs (122 KB PDF)

Figure S3 The Topology of the HC Network Is Sensitive to Deletion of Hubs (97 KB PDF)

Figure S4 Genetic Centrality of Date, Party, and Non-hubs (61 KB PDF)

Figure S5 Degree Correlation Pattern for a Reduced HC Network That Contains Only Nonessential Proteins (41 KB PDF)

Figure S6 Method Used to Create the HC Network (105 KB PDF)

Table S1 Fraction of Interactions with Shared GO Annotation (40 KB PDF)

Table S2 High Confidence Dataset of Multi-validated Protein Interactions (1.2 MB TXT)

## Abbreviations

ANCOVA: analysis of covariance
FYI: filtered yeast interactome
HC: high confidence
HTP: high throughput
LC: literature curated
