# Supplementary material for: Stratus Not Altocumulus: A New View of the Yeast Protein Interaction Network
Source: PLoS Biol. 2006 Sep 19;4(10):e317. doi: 10.1371/journal.pbio.0040317 (PMC1569888; doi:10.1371/journal.pbio.0040317)

Supplementary Figures

Supplementary Figure 1: Degree correlation for HC^h and HC^m networks

Degree correlations were computed as described [2]. HC^h is a subset of HC network with only HTP based multivalidated interactions. HC^m is a subset of HC network with only those interactions that are assessed via two different methods.

a) HC^h

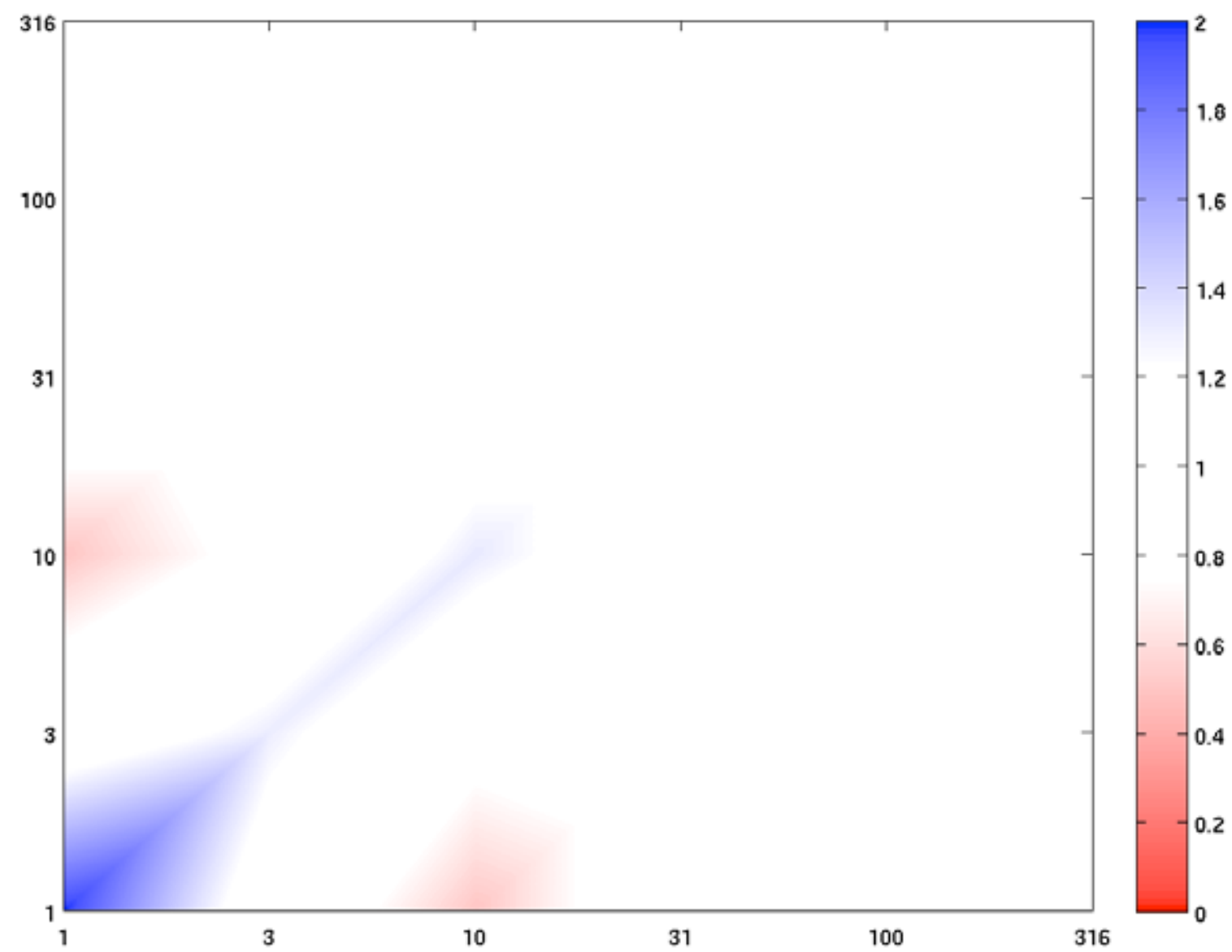

b) HC^m

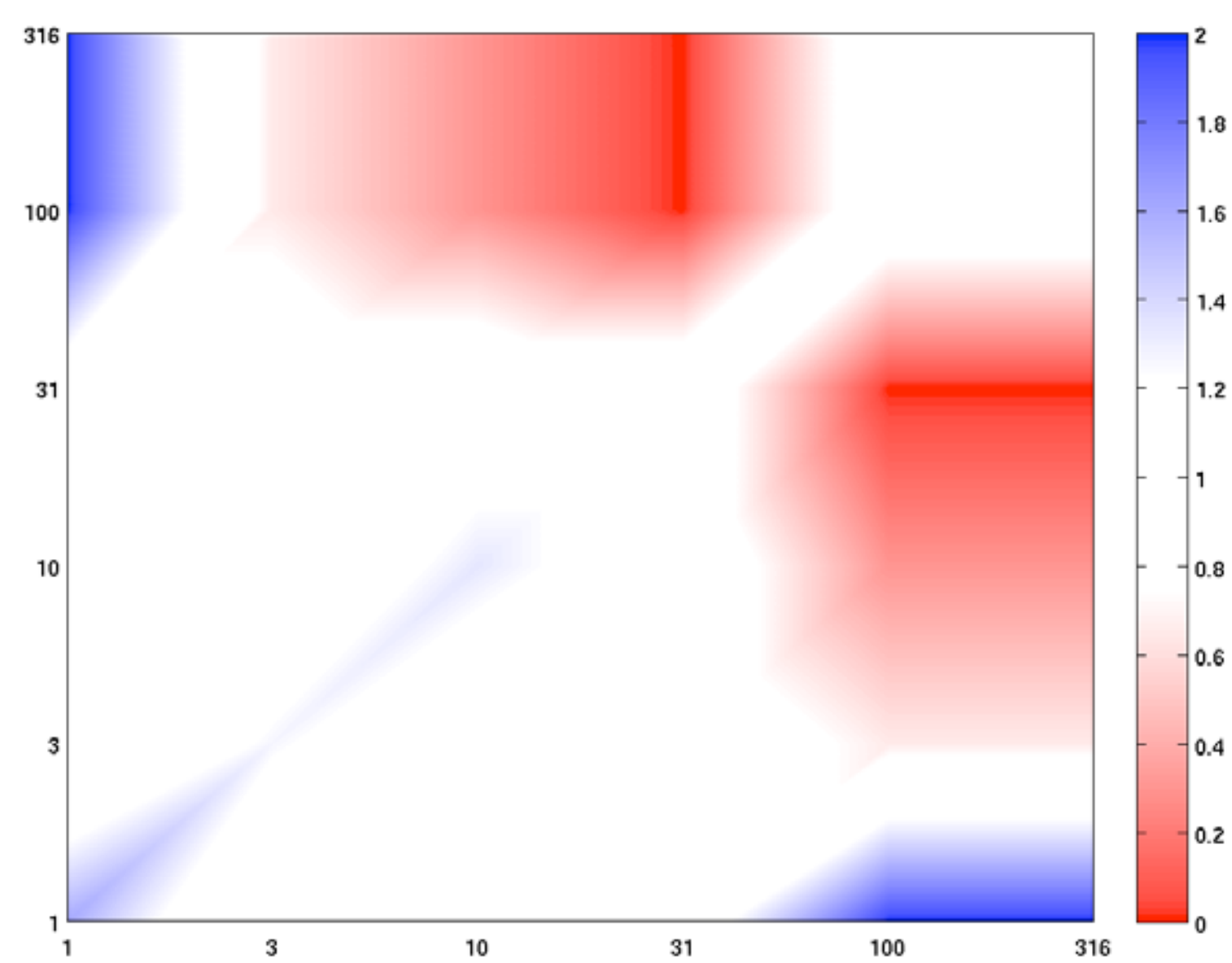

Supplement: Supplementary file 1 [file pbio.0040317.sg001.pdf]
