# Supplementary material for: Stratus Not Altocumulus: A New View of the Yeast Protein Interaction Network
Source: PLoS Biol. 2006 Sep 19;4(10):e317. doi: 10.1371/journal.pbio.0040317 (PMC1569888; doi:10.1371/journal.pbio.0040317)

Supplementary Figure 2: The topology of the LC and LC^{fyi} networks is not sensitive to deletion of hubs

Date or party hubs were successively deleted from either the FYI, LC^{fyi} or LC networks and the largest remaining component, as normalized to the size of the largest component prior to deletion, was used as a measure of residual network topology. Nodes were deleted serially in decreasing order of connectivity. Deletion of random nodes was used as a control. The LC^{fyi} network is based on the FYI date and party hub framework but with all interactions derived from the LC dataset. LC^{fyi} contains 4,111 interactions for the 1,231 proteins in common with FYI.

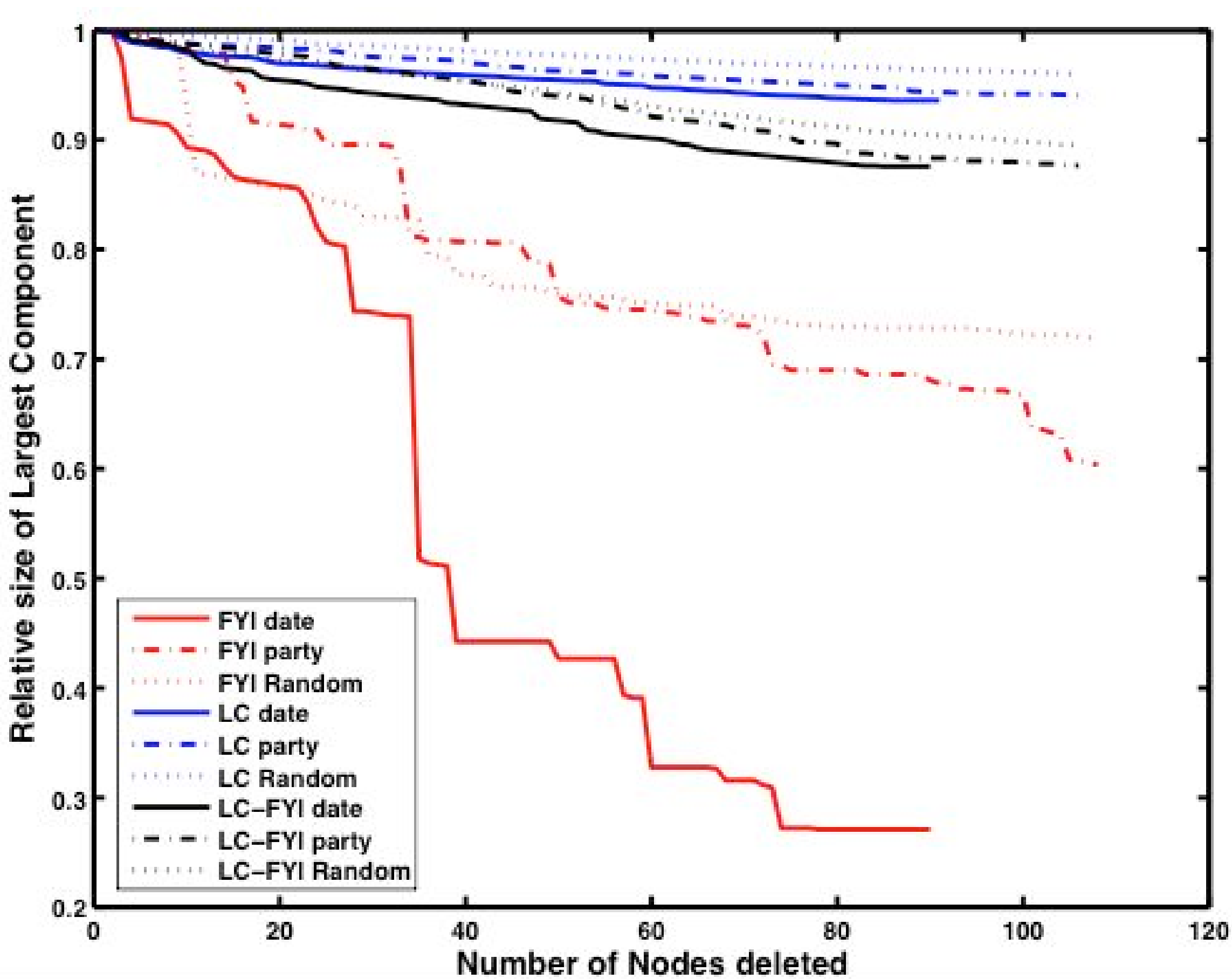

Supplement: Supplementary file 2 [file pbio.0040317.sg002.pdf]
