# Supplementary material for: Stratus Not Altocumulus: A New View of the Yeast Protein Interaction Network
Source: PLoS Biol. 2006 Sep 19;4(10):e317. doi: 10.1371/journal.pbio.0040317 (PMC1569888; doi:10.1371/journal.pbio.0040317)

Supplementary Figure 3: The topology of the HC network is sensitive to deletion of hubs

Plot shows the relative size of the largest component of the full HC network after deletion of the top 15 % of the hubs and, as a control, the same number of randomly picked proteins. In both cases, nodes were deleted serially in a descending order of connectivity. Results show that HC is indeed more sensitive to attack than random node failures, confirming previous conclusions [3].

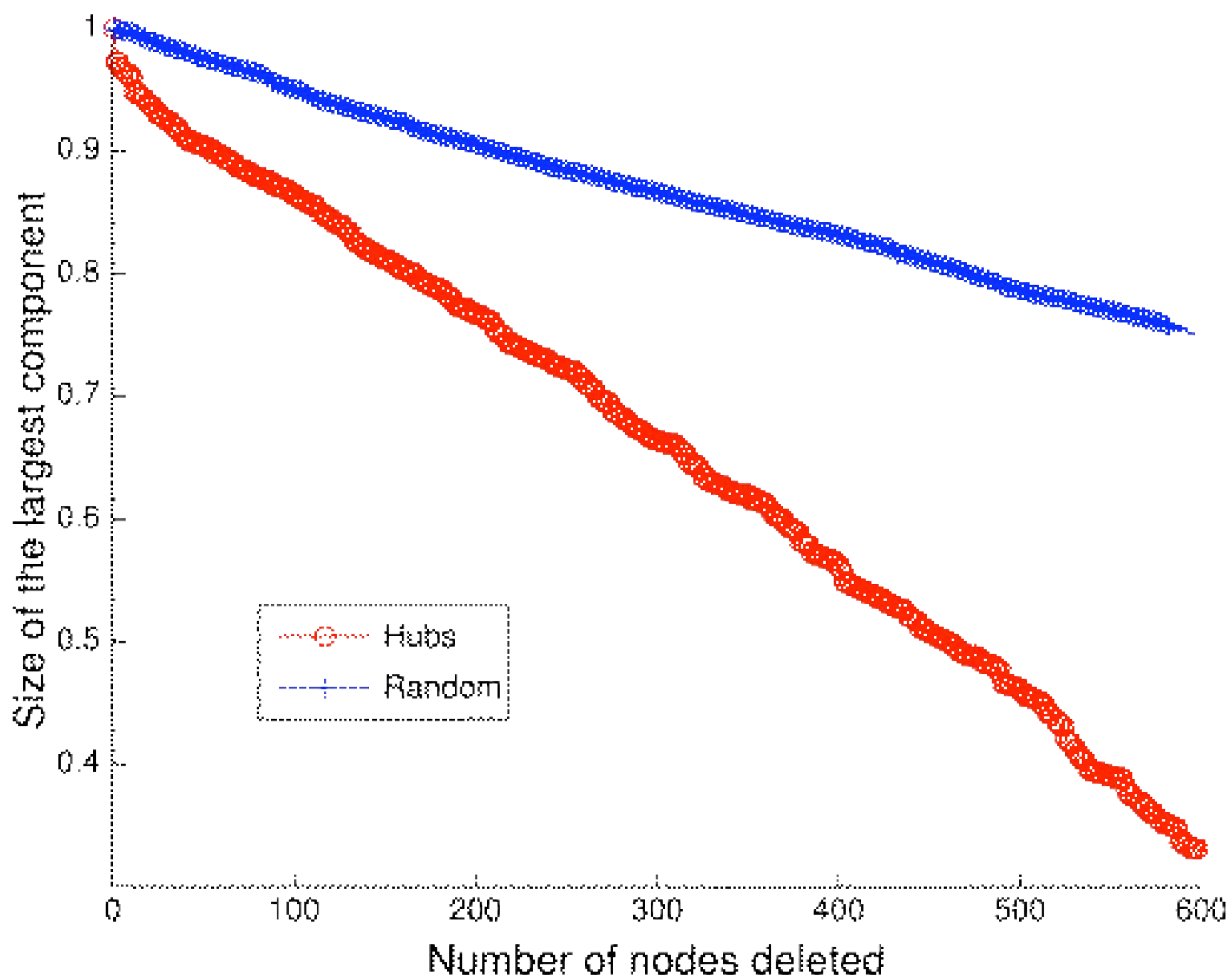

Supplement: Supplementary file 3 [file pbio.0040317.sg003.pdf]
