# Supplementary material for: Stratus Not Altocumulus: A New View of the Yeast Protein Interaction Network
Source: PLoS Biol. 2006 Sep 19;4(10):e317. doi: 10.1371/journal.pbio.0040317 (PMC1569888; doi:10.1371/journal.pbio.0040317)

Supplementary Figure 4: Genetic centrality of date, party and non-hubs

Date hubs show a higher degree of centrality in LC-GI genetic network, whereas party hubs are more connected in the HTP-GI genetic network.

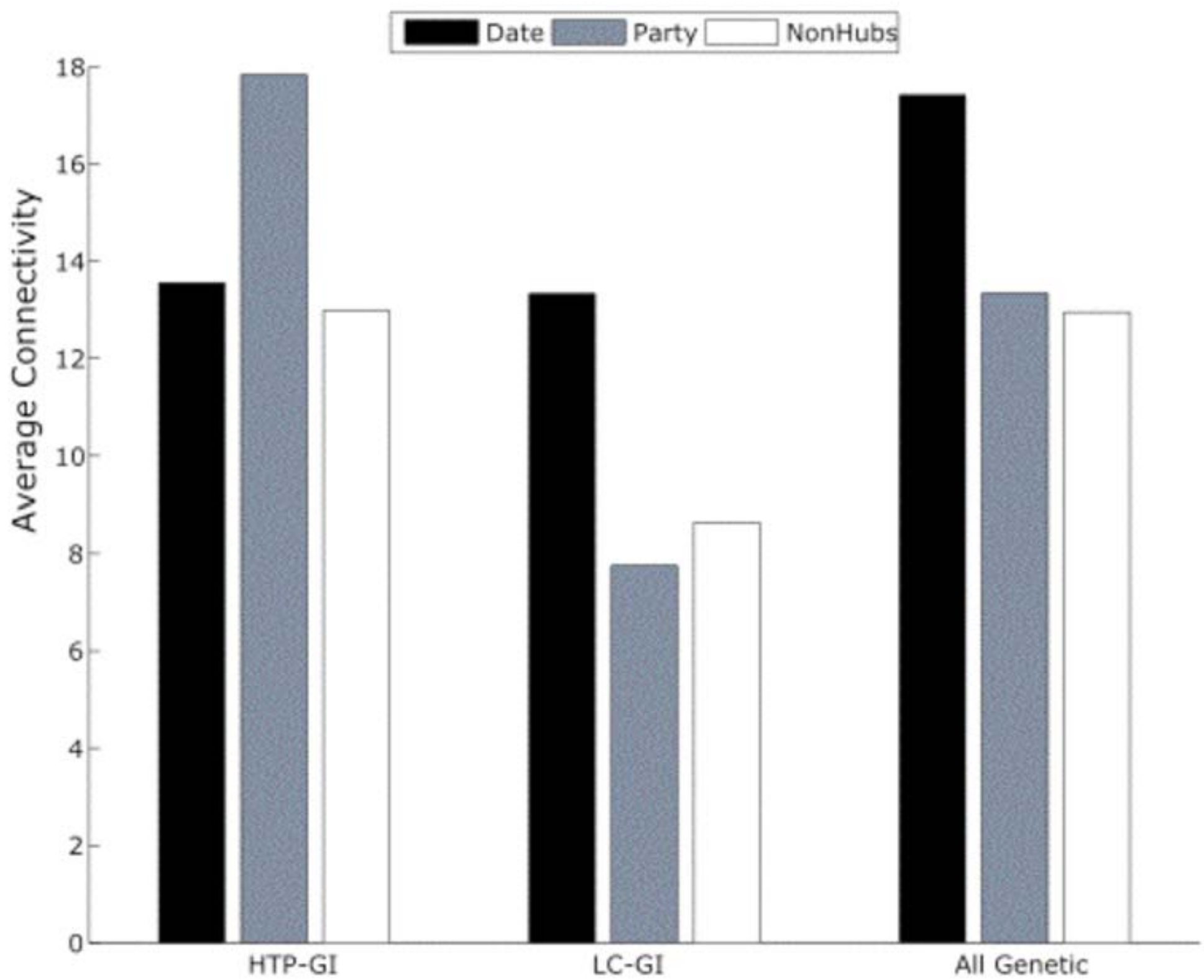

Supplement: Supplementary file 4 [file pbio.0040317.sg004.pdf]
