# Supplementary material for: Stratus Not Altocumulus: A New View of the Yeast Protein Interaction Network
Source: PLoS Biol. 2006 Sep 19;4(10):e317. doi: 10.1371/journal.pbio.0040317 (PMC1569888; doi:10.1371/journal.pbio.0040317)

Supplementary Figure 5: Degree correlation pattern for a reduced HC network that contains only non-essential proteins

Degree correlations were computed as described [2]. As the HC network is enriched in essentials relative to the HTP network, and essentials are more connected than non-essentials, the lack of degree anti-correlation shown in Figure 2 may result from enrichment of interactions among hubs in the HC dataset. We therefore regenerated the degree correlation figure for a reduced HC network in which only the interactions among non-essentials are retained. Anti-correlation was also not observed in this reduced network.

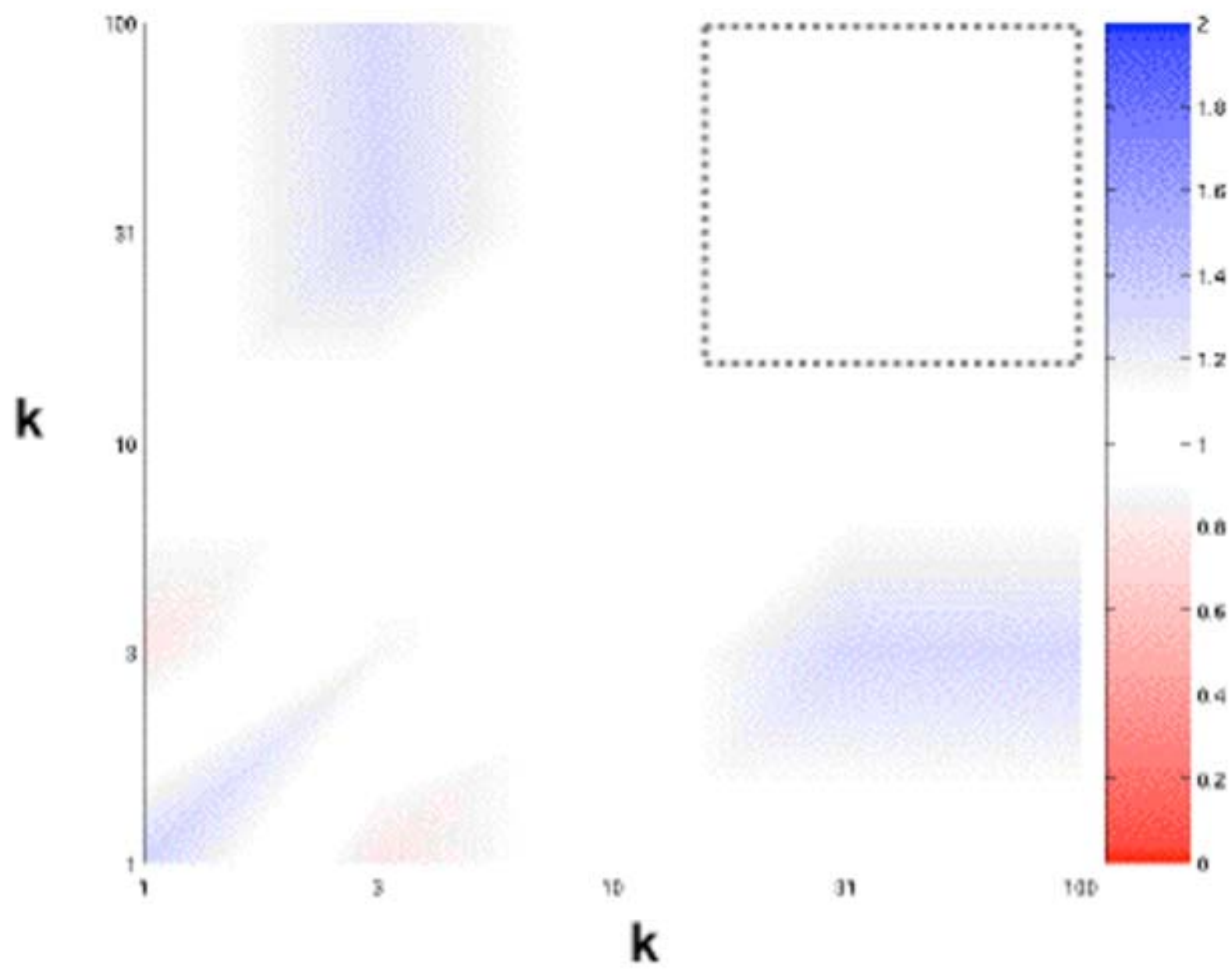

Supplement: Supplementary file 5 [file pbio.0040317.sg005.pdf]
