# Supplementary material for: Stratus Not Altocumulus: A New View of the Yeast Protein Interaction Network
Source: PLoS Biol. 2006 Sep 19;4(10):e317. doi: 10.1371/journal.pbio.0040317 (PMC1569888; doi:10.1371/journal.pbio.0040317)

Supplementary Figure 6: Method used to create the HC network

Schematic illustrates the procedure used to generate the high confidence (HC) network. Since independent efforts often curate data from the same papers, these common papers were removed from each of the curation sets and merged into the "Reduced" set. MV stands for multi-validated and SV stands for singly validated interactions. In all the networks, interactions were formed by linking baits to the preys in a minimal spoke model fashion [4].

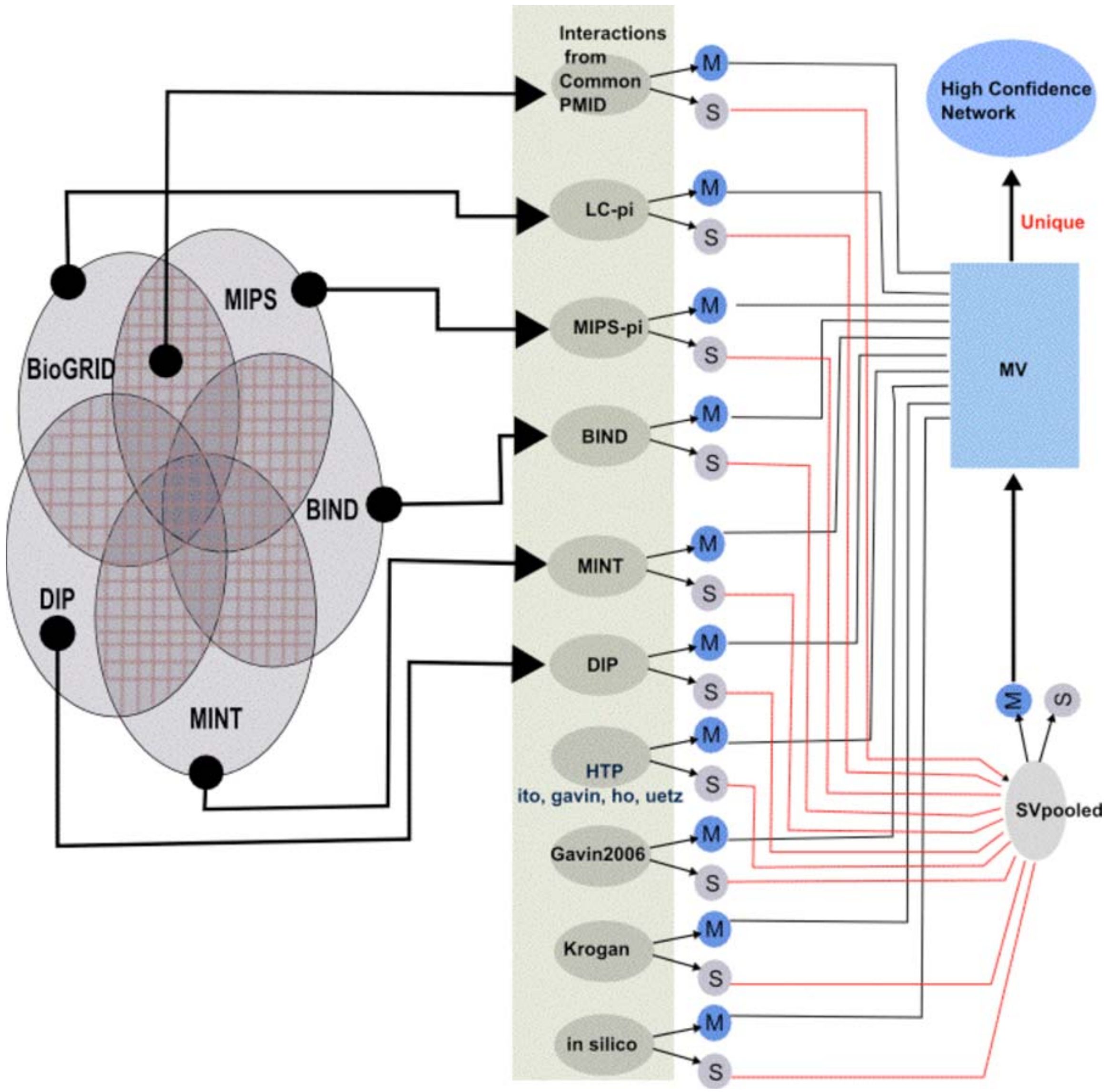

Supplement: Supplementary file 6 [file pbio.0040317.sg006.pdf]
