# Supplementary material for: Stratus Not Altocumulus: A New View of the Yeast Protein Interaction Network
Source: PLoS Biol. 2006 Sep 19;4(10):e317. doi: 10.1371/journal.pbio.0040317 (PMC1569888; doi:10.1371/journal.pbio.0040317)
Supplement: Supplementary file 7 [file pbio.0040317.st001.pdf]

Supporting Information for

Stratus not altocumulus: a new view of the yeast protein interaction network

Nizar N. Batada[1], Teresa Reguly, Ashton Breitkreutz, Lorrie Boucher, Bobby-Joe Breitkreutz, Laurence Hurst[3] and Mike Tyers[1,2]

[1] *Samuel Lunenfeld Research Institute, Mount Sinai Hospital, 600 University Ave, Toronto, Canada M5G 1X5*
[2] *Department of Medical Genetics and Microbiology, University of Toronto, Toronto, Canada M5S 1A8*
[3] *Department of Biology and Biochemistry, University of Bath, Bath, UK, BA2 0TA*

Supplementary Table

Supplementary Table 1: Fraction of interactions with shared GO annotation.

Shared annotation for interacting pairs in the FYI [1] and the full HC network were calculated. Statistical significance of differences in the fraction of shared annotation between FYI and HC were computed using Fisher's Exact Test ($P<0.01$). Only those interactions are considered in which both protein partners have a known GO annotation.

	GO Function	GO Process	GO Component
**FYI**	0.62	0.67	0.63
**HC**	0.43	0.43	0.49
**HC^h**	0.52	0.50	0.52
**HC^m**	0.49	0.48	0.55
**LC**	0.37	0.36	0.46
